# Exploring Epigenetic Ageing Using Direct Methylome Sequencing

**DOI:** 10.3390/epigenomes9030025

**Published:** 2025-07-14

**Authors:** Elena-Cristina Găitănaru, Roua Gabriela Popescu, Andreea-Angelica Stroe, Sergiu Emil Georgescu, George Cătălin Marinescu

**Affiliations:** 1 Independent Research Association, 012416 Bucharest, Romania; cristina.gaitanaru@independent-research.ro; 2 Blue Screen SRL, 012416 Bucharest, Romania; 3 Department of Biochemistry and Molecular Biology, Faculty of Biology, University of Bucharest, Splaiul Independenței 91–95, 050095 Bucharest, Romania; angelica-andreea.stroe@bio.unibuc.ro (A.-A.S.); sergiu.georgescu@bio.unibuc.ro (S.E.G.)

**Keywords:** DNA methylation, nanopore epigenetic clock, nanopore adaptive sampling, epigenetic clock, ageing related CpGs

## Abstract

**Background/Objectives**: Advances in nanopore sequencing have opened new avenues for studying DNA methylation at single-base resolution, yet their application in epigenetic ageing research remains underdeveloped. **Methods**: We present a novel framework that leverages the unique capabilities of nanopore sequencing to profile and interpret age-associated methylation patterns in native DNA. **Results**: Unlike conventional array-based approaches, long reads sequencing captures full CpG context, accommodates diverse and repetitive genomic regions, removes bisulfite conversion steps, and is compatible to the latest reference genome. **Conclusions**: This work establishes nanopore sequencing as a powerful tool for next-generation epigenetic ageing studies, offering a scalable and biologically rich platform for anti-ageing interventions monitoring and longitudinal ageing studies.

## 1. Introduction

Ageing is a multifaced biological process characterized by the progressive accumulation of molecular alterations in cells and tissues, ultimately leading to physiological decline and increased susceptibility to diseases such as cancer, cardiovascular disorders, and neurodegeneration [1,2,3,4]. Although phenotypic signs like grey hair and skin atrophy are commonly associated with ageing, in fact, the first signs appear within cells long before they become visible [5], as inner molecular events, including telomere shortening [6,7,8,9], production, and the accumulation of free radicals through mitochondrial dysfunction [10], “inflammageing” manifested by increased expression of senescence-associated secretory phenotype (SASP) factors [11,12,13], DNA damage, protein balance loss, deregulated nutrient sensing, and altered cellular communication [14].

Currently, in the context of population ageing, it is of great interest to develop different solutions that extend lifespan but also maintain the health of the organism. Studies on ageing are focused on epigenetic modifications, especially DNA methylation (DNAm), as it is an easily quantifiable marker using epigenetic clocks [15,16,17,18], but also due to the success of epigenetic reprogramming in model organisms, representing a real potential anti-ageing treatment [1,4,19,20,21,22,23,24,25]. The DNAm patterns (referred to as the “*methylome*”) are established and subsequently preserved through cell divisions to maintain cellular identity [26]. DNAm has been highlighted as a potential biomarker of ageing due to the variability influenced by chronological age, more specifically represented by a global hypomethylation wave accompanied by hypermethylation of CpG sites that physiologically would be unmethylated [27,28,29,30].

The correlation of methylome and chronological age was extensively studied during the last decade, leading to predictors of biological age based on CpG methylation patterns alone. These predictors are generally referred to as “*epigenetic clocks*” [15,16,31,32,33,34,35]. These are believed to be accurate for any tissue across the entire life course, linking the development and maintenance processes to biological ageing into “epigenetic clock theory of ageing” [34].

The currently available epigenetic clocks, such as those developed by Hannum et al. [15], Horvath [16], and Weidner et al. [17], are based on Illumina that use bisulfite-converted DNA. However, these methods have critical limitations: they cannot distinguish between 5-methylcytosine (5mC) and 5-hydroxymethylcytosine (5hmC) [36], and they have limited resolution in highly repetitive sequences or non-coding genomic regions. In contrast, Oxford Nanopore Technologies (ONT) offers several advantages, including real-time, direct sequencing of native DNA with long reads, enabling the detection of both 5mC and 5hmC and improved alignment across repetitive and non-coding genomic regions, with a more comprehensive view of epigenetic modifications.

Therefore, given the past limitations and the current possibilities to understand the dynamics of DNA methylation patterns and how it changes or correlates with chronological age, the aim of this study was to propose a direct epigenome sequencing workflow on ONT technology, as a tool for future ageing research. To address this, we analyzed seven DNA samples ranging from people 5 to 91 years old, proposing a framework to capture age-dependent methylation variation across the lifespan.

## 2. Results

To uncover the dynamic of DNA methylation in the ageing process, we sequenced human genomic DNA with Nanopore’s adaptive sampling technology. From the seven aggregated bedMethyl files we identified a total of 64,390,996 CpG sites. Among these CpGs, by correlating the chronological age with the methylation frequencies, also known as β-values, using two types of linear regression models, we observed modifications in the level of 5mC and 5hmC that were highly correlated with age (R^2^ ≥ 0.8, *p*-value < 0.001). The exact CpG datasets can be found in Supplementary Material S1, Tables S1–S3.

### 2.1. Enrichement of Genomic Target Regions with Adaptive Sampling

To test the performance of the adaptive sampling technique we used our adaptive sampling panel that contained a total of 17,978 target regions across the human genome (assembly GRCh38) with sizes ranging from approximately 8 kb to 888 kb, totaling around 327 Mb. The created adaptive sampling panel can be found as a .bed file in Supplementary Material S2.

By sequencing seven human genomes of different ages, we obtained a total of 79.63 Gb and 114.94 M reads (Supplementary Material S1, Table S4), with a mean length of on-target fragments (N50) of 8.5 kb. DNA fragments that did not align with the panel regions were ejected at around 500 bp. Overall, the adaptive sampling method managed to achieve a median enrichment ranging from 2.42X to 4.35X in the defined target regions, relative to off-target regions.

At the end of the sequencing runs, we observed a variation in reads number, ranging from 9.11 M to 27.1 M. This variation was caused not only by differences in the initial DNA input for each sample, which automatically induced differences in the number of femtomoles loaded into each R10.4.1 flow cell, but also to variations in the number of available pores of each new flow cell. By the end of the first run (S46) we noticed that the number of reads generated was low and the enrichment was not as notable. We deduced that the root of the problem was the initial input of DNA (2 µg), which was the recommended yield in the Ligation protocol for RRMS [37]. Additionally, we observed an approximately 25% loss of DNA by the end of the library preparation step. This eventually led to loading around 30 femtomoles of DNA for this sample, which is far less than the 50–65 femtomoles recommended in the adaptive sampling documentation file [38]. To achieve the advised molarity, for the next six samples, the initial input of DNA was increased to 3–4 µg.

**Figure 1:**
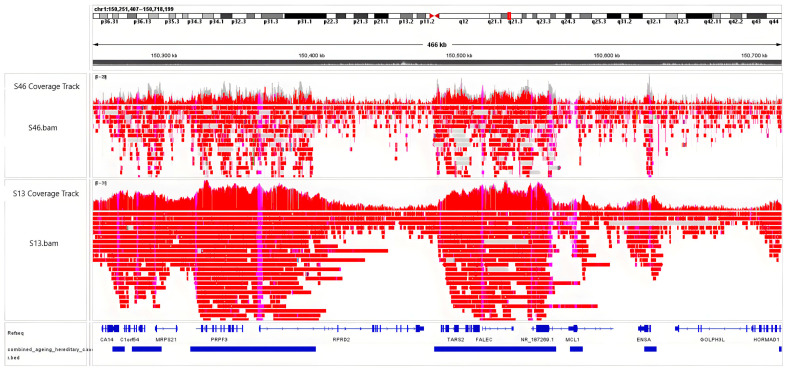
IGV snapshot of ONT’s adaptive sampling enrichment in two samples. The top track shows enrichment and coverage for sample S46, prepared with a 2 µg DNA input. The bottom track shows the enrichment and coverage for sample S13, which used a 4 µg of DNA input.

An IGV snapshot comparing two samples (Figure 1) shows the coverage difference between sample S46, which had an initial input of DNA of 2 µg for the first step of library preparation, and sample S13, which used 4 µg. Different levels of enrichment can be observed in the coverage tracks, reflecting how DNA input affects the adaptive sampling Nanopore sequencing coverage. The full overview of DNA inputs and sequencing runs can be found in Supplementary Material S1, Tables S4 and S5.

As reported previously by other researchers using different panels [39,40], ONT’s adaptive sampling technique effectively rejected DNA fragments outside the target regions and enriched those of interest.

### 2.2. The DNA Methylation Landscape Across the Lifespan

By aggregating the seven DNA methylation datasets, we identified a total of 64,390,996 CpG sites. The data were further filtered for minimal sequencing depth of five reads. Linear regression was then applied (see Section 2.3) and the results were further filtered by regression statistics (*p* value < 0.05, R^2^ ≥ 0.8). Only complete datasets, which had methylation values for all seven age points, were considered for further analysis. We grouped the datasets based on the type of DNA modification, aiming to observe the dynamics of 5mC and 5hmC, respectively. After splitting the filtered data, we obtained 15,620 CpGs in the 5mC file and 6397 CpGs in the 5hmC file (Supplementary Material S1, Tables S1 and S2).

**Figure 2:**
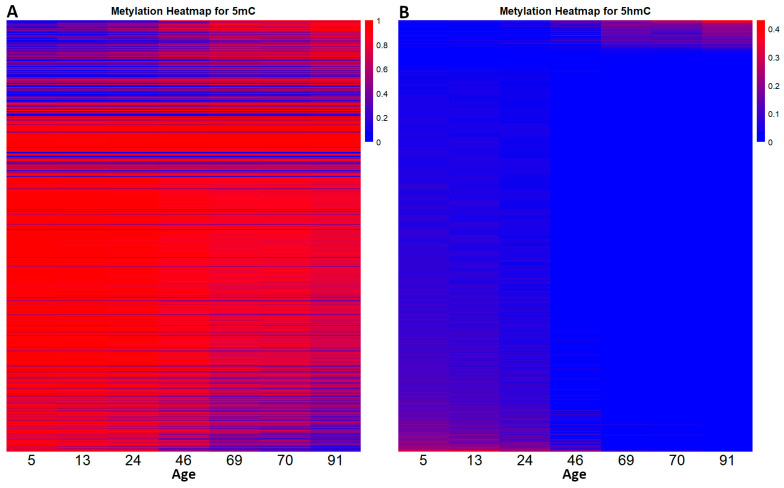
The dynamic relationship between DNA methylation and DNA demethylation throughout the human lifespan. Heatmap (**A**) shows the level of DNA methylation (5mC variation) for each CpG in the 5mC dataset at different ages. Heatmap (**B**) outlines the level of 5hmC variation from the CpGs in the 5hmC dataset at different ages. In this case red indicates an increased level of 5hmC.

With the intention of seeing how the DNA methylation landscape changes as an individual grows old, we distributed the β-values, from all the CpGs in the two datasets using heatmaps (Figure 2).

By following the distribution, we can see that DNA methylation does not follow a global pattern. In the 5mC dataset, some CpGs either get hypomethylated (73.75%) or hypermethylated (26.25%). The same phenomenon can be observed in the 5hmC dataset, where 80.65% of CpG sites lose 5hmC and 19.35% gain 5hmC. In both datasets, some CpGs remained unchanged. Also, the methylation landscape in the developmental years (0–20 years) appears to be very stable. From age 24 to 91 we observed a gradual loss of 5mC, respectively, a gradual gain of 5hmC.

### 2.3. Detecting Age-Related DNA Methylation Changes with a Simple Linear Regression

**Figure 3:**
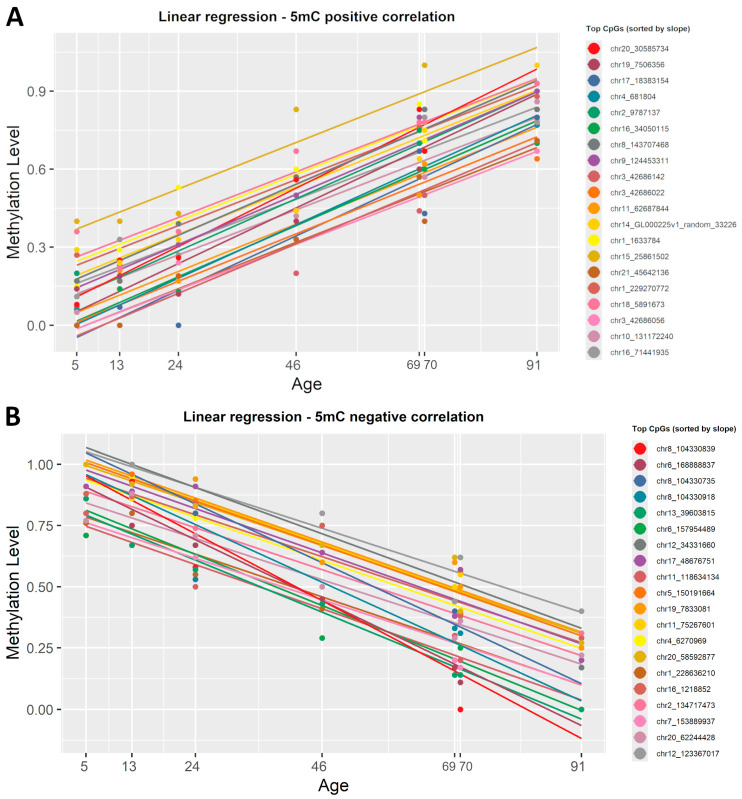
Linear regression lines showing the correlation between 5mC β-values and chronological age. (**A**) Colored lines show the first 20 CpG sites, sorted by slope, that presented a positive correlation with age. (**B**) Colored lines show the 20 most representative CpG sites that have a negative correlation with age. Each regression line corresponds to a CpG site, and each point is determined by chronological age and β-values. All identified CpG sites showed a significant linear correlation with age (R^2^ ≥ 0.8, *p* < 0.001).

To identify which CpG sites undergo significant modifications with age, we applied a simple linear regression in R (version 4.4.1) on the 5mC dataset. To start, β-values were correlated with the chronological age. This regression yielded a slope value, correlation coefficient R^2^, and *p*-value for each CpG site present in the dataset. The data were then sorted by slope to highlight top CpGs directly and inversely correlated with ageing (Supplementary Material S1, Table S1). In the 5mC dataset, 535 CpG sites were statistically significant (R^2^ ≥ 0.8, *p* < 0.001) positively correlated with age, while 1794 CpG sites showed a statistically significant (R^2^ ≥ 0.8, *p* < 0.001) negative correlation. We created a list, sorted by the slope value, with the first 20 CpGs that were either positively or negatively correlated with age. Figure 3 shows the plotted correlation between β-values and chronological age at these genomic locations.

To highlight the strongest correlation with age in this dataset, we also sorted the list of CpGs by correlation coefficient. This revealed that chr22:48462175 (R^2^ = 0.99) showed the strongest negative correlation with chronological age, whereas chr10:42898383 (R^2^ = 0.99) showed the strongest positive correlation with age.

### 2.4. Detecting Age-Related DNA Methylation Changes with ElasticNet Regression

**Figure 4:**
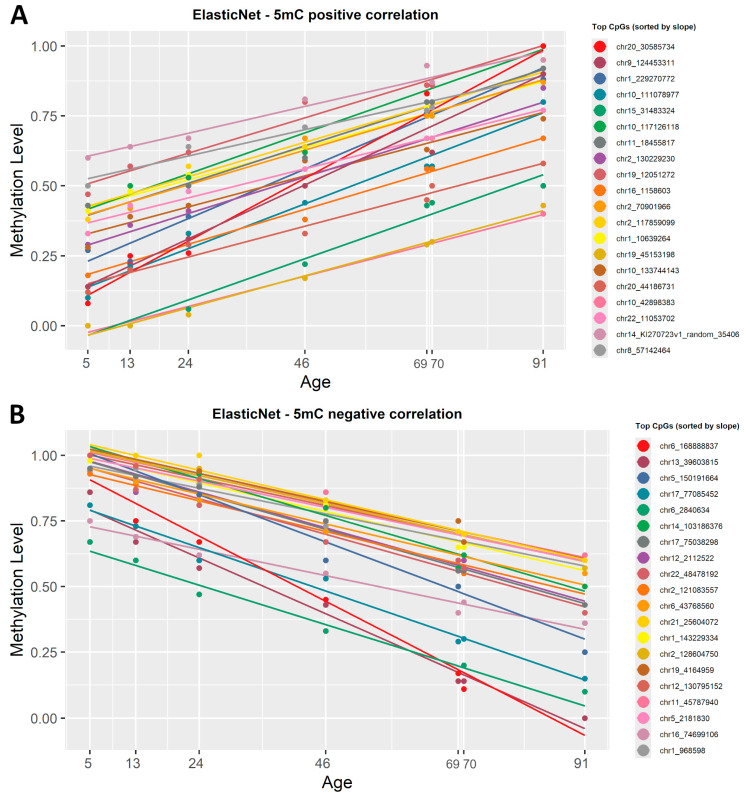
ElasticNet regression lines showing the correlation between 5mC β-values and chronological age. (**A**) The first 20 CpG sites, sorted by slope in descending order, that show a positive correlation with age. (**B**) The 20 most representative CpG sites that show a negative correlation with age, based on ascending slope values. Each regression line corresponds to a CpG site and each point corresponds to a chronological age. All CpG sites identified have a significant correlation with age (R^2^ ≥ 0.8, *p* < 0.0005).

ElasticNet regression, previously used in the construction of epigenetic clocks, was applied. Being a penalized regression model, ElasticNet automatically selected the most representative CpGs based on setting R^2^ > 0.92 and *p*-value < 0.0005. From the 5mC dataset, 108 CpG sites were automatically picked as being highly correlated with age, 43 CpG sites were positively correlated and 148 were negatively correlated (Supplementary Material S1, Table S3). The list of CpGs was then sorted by slope and plotted in Figure 4.

To see if there is a difference between the filtered simple linear regression and ElasticNet models, we also sorted the list by correlation coefficient (R^2^) and observed no difference when it comes to the CpG sites with the strongest negative or positive correlation with age.

### 2.5. Detecting Age-Related Changes in the Level of 5hmC with Linear Regression

**Figure 5:**
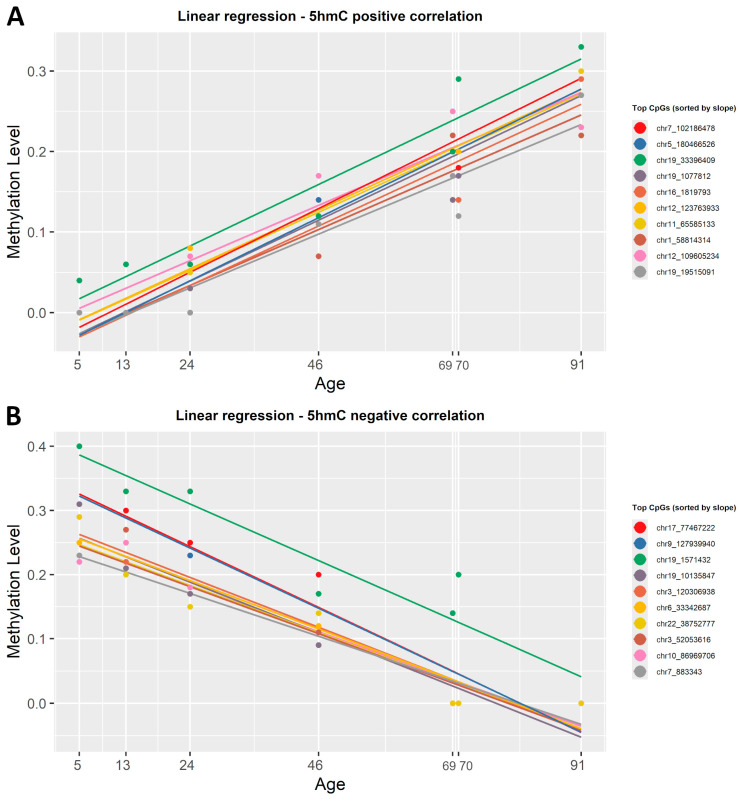
Linear regression lines showing the correlation between 5hmC β-values and chronological age. (**A**) Colored lines show the first 20 CpG sites, sorted by slope, that presented a positive correlation with age. (**B**) Colored lines show the 20 most representative CpG sites that have a negative correlation with age. Each regression line corresponds to a CpG site, and each point is determined by chronological age and β-values. All identified CpG sites showed a significant linear correlation with age (R^2^ ≥ 0.8, *p* < 0.001).

To identify which CpG sites change in the level of 5hmC with age, we correlated the β-values in this dataset with the chronological age using both regression methods mentioned above. After applying the simple linear regression function, 563 CpGs out of the 6397 analyzed were positively correlated with age, while 5834 showed a negative correlation (Supplementary Material S1, Table S2). The strongest gain in 5hmC with age was observed at chr12:123763933 (R^2^ = 0.98) for positive correlation, whereas the most significant loss occurred at chr21:8390848 (R^2^ = 0.98) for negative correlation. Figure 5 shows the correlation between β-values and chronological age for the top 20 CpG sites in the 5hmC dataset, sorted by slope. When applying ElasticNet regression to the 5hmC dataset, no CpG sites were selected as significantly associated with age.

### 2.6. Exploring New Methylation Markers and Their Genomic Locations

We used the sorted CpGs resulted from all the regression datasets. This way a total of 2610CpG sites that passed the filters (R^2^ ≥ 0.8, *p* < 0.001) resulted, including 2329 CpGs from the 5mC linear regression dataset (535 positively and 1794 negatively correlated with age), 90 CpGs from the 5hmC linear regression dataset (49 positively and 41 negatively correlated with age), and 191 CpGs from the 5hmC ElasticNet dataset (43 positively and 148 negatively correlated with age).

**Figure 6:**
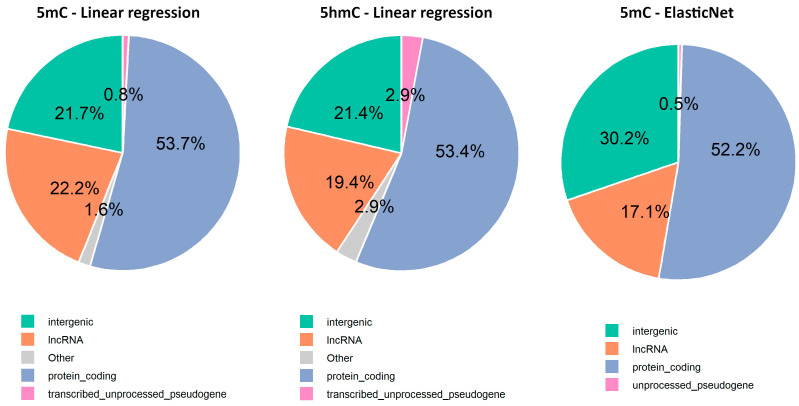
Repartition of age-related CpG sites across the top four genomic regions identified by Nanopore adaptive sampling direct methylation sequencing.

**Figure A1:**
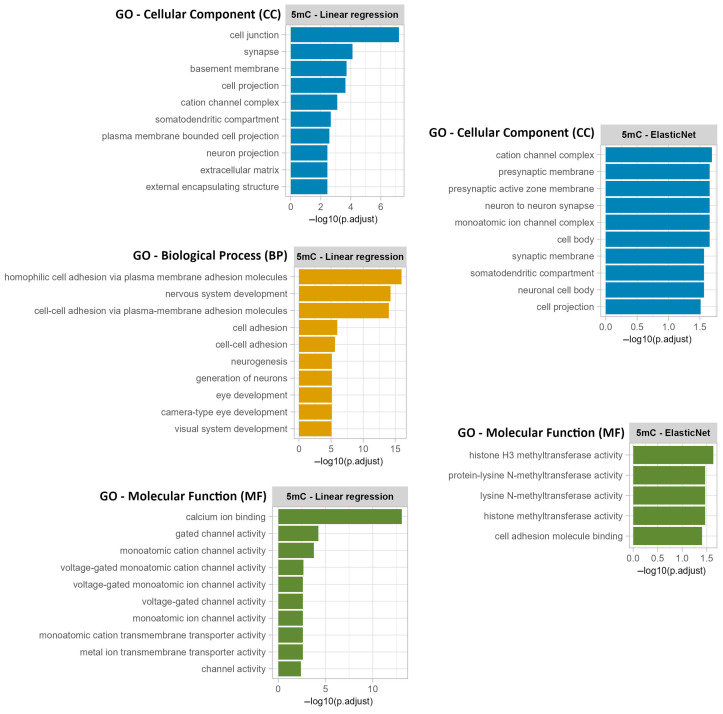
Gene Ontology (GO) analysis performed using CpG sites from intragenic regions from the 5mC linear regression dataset and 5mC ElasticNet dataset.

The gene annotation analysis revealed that 75.6% of the selected CpGs were in intragenic regions, while only 24.4% were in intergenic regions (Supplementary Material S1, Table S6). Among these, most were associated with protein-coding genes, followed by long non-coding RNAs (lncRNAs), transcribed unprocessed pseudogenes, and unprocessed pseudogenes (Figure 6). Additionally, Gene Ontology analysis performed using CpG sites from intragenic regions suggests age-related changes in cellular components (CC), biological processes (BP), and molecular functions (MF), particularly related to the extracellular matrix, ion channels, development, histone modification, and protein methyltransferase activity (Figure A1, Supplementary Material S1, Table S7).

We found a total of 9 CpGs overlapping with the CpGs used in four established epigenetic clocks: 3 with Horvath’s clock (353 CpGs), 7 with Hannum’s clock (71 CpGs), none with Weidner’s clock (102 CpGs), and 6 with Horvath’s Skin & Blood clock (391 CpGs). (Table 1, Supplementary Material S1, Table S8).

## 3. Discussion

This study proposes an epigenetic ageing framework using ONT’s adaptive sampling technique for DNA methylation detection and epigenetic clocks training. With our adaptive sampling panel, we were able to identify 64,390,996CpG sites in human genomic DNA extracted from blood samples. To our knowledge, no previous study reported the finding of such large number of CpG sites. We presume this high number is the result of long reads, which enable mapping of highly repetitive regions, known to be challenging for short reads or array-based platforms. This technique enabled the detection of potentially new methylation biomarkers that take into consideration not only 5mC, but also 5hmC DNA modification, known as the first step in the demethylation process [41]. The list of CpGs that we identified as having a strong correlation with age (R^2^ ≥ 0.8) could be used as a template for constructing future epigenetic clocks once more long-read genomic data are available (Supplementary Material S1, Tables S1–S3). Our framework is ready to be used, adapted, and refined as it is publicly accessible. The framework can be used for complete genome PromethION flow cells, but also shows promising results on MinION, smaller and less expensive flow cells, using adaptive sample.

After assessing the first sequencing run, we noticed that the Oxford Nanopore recommended DNA input is not sufficient to produce enough reads for a good enrichment of target regions. This impediment was also previously observed by Yuen et al. [39]. Although the required amount of DNA for this kind of adaptive sequencing is double the required quantity for whole genome sequencing (1 µg) [37], even 2 µg of DNA is not enough for this technique. Therefore, the DNA input quantity for some samples was doubled to obtain the recommended amount of femtomoles (50–65 femtomoles) that needed to be loaded in the flow cell at the end of the library preparation step (Figure 1). While this does not represent a limitation for a future epigenetic clock, which is used in clinical conditions to either estimate someone’s biological age or to evaluate the outcome of different anti-ageing strategies, it represents a limitation in the forensics context where the sample is limited [40].

To find which CpG sites were significantly correlated with chronological age, we tested two important statistical approaches. The first one was the simple linear regression previously used in forensic DNA methylation studies [39,40,42,43,44,45,46,47] (Supplementary Material S1, Tables S1 and S2). The second was the ElasticNet regularized regression model (Lasso and Ridge) [48], the preferred method for epigenetic clock construction, previously used in clocks, such as Hannum [15], Horvath [16], Weidner [17], and Skin & Blood [49] (Supplementary Material S1, Table S3). We observed no significant differences between the two regression models; both identified the same top significant CpG sites that showed either a positive or negative correlation with chronological age (Figure 3, Figure 4 and Figure 5). Overall, both regression models managed to find a good number of CpGs highly correlated with age (R^2^ ≥ 0.8, *p*-value < 0.001). A notable difference is that ElasticNet automatically selected the best CpGs from the dataset, while for the simple linear regression, we had to manually sort by slope, with a higher slope meaning better resolution for the ageing clock.

Contrary to others’ findings, the most significant CpGs correlated with age were not located in age-related genes [40,50], not even in any of the 307 genes from the GenAge database [51]. This is mainly due to the panel that was used and the coverage filter (reads > 5) in areas where these age-related genes were located.

As Kenneth Raj and Steve Horvath previously noted [52], the molecular mechanisms driving age-associated DNA methylation changes remain elusive. The assumption that methylation directly silences genes or promoter regions by altering transcription does not appear to hold consistently. Correlating age-related CpG methylation with specific gene loci has not yielded meaningful results so far. Notably, most of the age-associated CpGs identified using nanopore-based direct methylation sequencing are in intragenic regions. However, consistent with Horvath’s observation that age-related methylation changes are tightly linked to developmental pathways, we identified four 5mC sites significantly correlated with age in exon 1 of the KLHL40 gene. This gene encodes a substrate-specific adaptor of the BCR (BTB-CUL3-RBX1) E3 ubiquitin ligase complex, previously reported to play a key role in skeletal muscle development [53]. Given that sarcopenia, the progressive loss of skeletal muscle mass and function, is a major hallmark of ageing, the presence of age-correlated methylation changes in KLHL40 supports the hypothesis that epigenetic drift with age may impact key developmental and maintenance pathways in muscle tissue. These findings raise the possibility that specific intragenic methylation changes, although limited in number, may have functional consequences in tissue-specific ageing phenotypes. Future studies combining transcriptomic and proteomic data with methylome profiling may point to the mechanistic relevance of such methylation marks, particularly in the context of age-related diseases like sarcopenia.

Moreover, Gene Ontology (GO) enrichment analysis of CpG sites located within intragenic regions revealed a strong association with age-related processes involved in neurodevelopment, ion transport, and structural cell organization (Figure A1, Supplementary Material S1, Table S7). A predominant number of terms clustered around nervous system development, neurogenesis, neuron differentiation, and brain morphogenesis, highlighting the well-established connection between epigenetic modifications and neuronal ageing. Additional enriched terms pointed to sensory organ development, particularly eye and auditory system formation, including camera-type eye development, retina development, and auditory receptor cell morphogenesis, suggesting that age-related methylation changes may affect both central and peripheral sensory functions. Multiple GO terms were also associated with extracellular matrix organization, cell adhesion, and actin cytoskeleton components (e.g., collagen-containing extracellular matrix, focal adhesion, and actin filament bundle), showing potential age-related changes in tissue integrity and intercellular signaling. Furthermore, enriched molecular function terms such as voltage-gated calcium channel activity, monoatomic ion transmembrane transporter activity, and ligand-gated cation channel activity pointed out shifts in ion channel dynamics, which are critical for cellular excitability, particularly in neurons and muscle cells.

These findings suggest that other genes outside of the ageing gene database [51] could play a role in ageing process, and the CpG sites identified in them could be potential new DNA methylation biomarkers. These biomarkers could be used for constructing future epigenetic clocks. In the history of epigenetic clocks, bisulfite treatment and Illumina Infinium platforms were the preferred methods for DNA methylation detection. Currently, this technology is outperformed, because these platforms contain a limited number of CpG sites that can be identified. As the Infinium arrays were built on hg18 and hg19 reference genomes, to compare previous work in the field with the most recent genome builds, CpG site coordinates must be converted, a process that we implemented in our custom R code. This conversion step, however, can result in the loss of some CpG sites that do not align with the updated reference genome. Our publicly available code includes this conversion process. A small number (9) of our identified CpGs most correlated with ageing overlapped with those include in the construction of established clocks such as Hannum [15], Horvath [16], Weidner [17], or the Skin & Blood [49] clock (Supplementary Material S1, Table S8).

Despite several limitations of the current study, including the absence of R10.4.1-based human DNA methylation datasets in existing repositories and the relatively small sample size (*n* = 7) in our own sequencing cohort, this work presents a robust and user-friendly framework. The pipeline is readily adaptable to various nanopore sequencing methods and devices and can be applied to other datasets as more data become available. This will enable researchers to replicate the workflow on their own data, estimate biological age from human blood samples, and develop a multi-tissue epigenetic clock using long-read nanopore sequencing data.

## 4. Materials and Methods

### 4.1. Sample Collection and DNA Extraction

Genomic DNA was extracted from seven anonymized samples consisting of leftover blood from routine clinical procedures obtained from HemoLab Clinic (Bucharest, Romania) from people 5 to 91 years old. A complete demographic of the cohort is presented in Supplementary Material S1, Table S9. DNA was extracted from these blood samples using two methods. Initially, DNA was extracted using Suguna et al. [54] genomic DNA isolation from human whole blood protocol following the authors’ instructions, with some adjustments in the precipitation of DNA step. In brief, isopropanol and 70% ethanol were previously kept at −20 °C and on ice during pipetting in each Eppendorf tube containing the DNA supernatant. To maximize the DNA precipitation, the Eppendorf tubes containing the precipitated DNA were also kept at −20 °C for up to an hour and then towed at 4 °C. Moreover, DNA was rehydrated in 25 µL of Milli-Q water, instead of 50 µL TE buffer. In addition, the Wizard^®^ Genomic DNA Purification Kit (TM050, Promega, Madison, WI, USA) was also used to extract DNA according to the manufacturer’s protocol. After 24–48 h, DNA concentration was measured with the NanoDrop ND−1000 Spectrophotometer (Thermo Scientific, Waltham, MA, USA) by reading light absorption at 260 nm and DNA purity was evaluated by the reports of light absorption at 260 nm/280 nm (A260/280) respective to 260 nm/230 nm (A260/230).

### 4.2. DNA Purification and Fragmentation

Since the A260/280 ratios were not in the range of 1.8–2 for the DNA extracted with the Suguna et al. [54] protocol, the samples were further purified using the DNeasy PowerClean Pro Cleanup Kit (12997−50, Qiagen, Venlo, The Netherlands) as per the manufacturer’s recommendations. Additionally, to prevent pore clogging, the extracted and purified DNA was sheared using a 29-gauge blunt needle connected to a 1 mL syringe. The DNA shearing process consisted of placing the needle close to the bottom of the tube and passing the eluted DNA 20 times through it, making sure to minimize creation of air bubbles. Fragment size distribution ranging from 6 to 20 kb was confirmed on 0.5% agarose gel electrophoresis.

### 4.3. Adaptive Sampling Target Panel Assembly

The adaptive sampling panel used for this experiment was established by merging the reduced representation methylation sequencing (RRMS) panel with the hereditary cancer panel [55]. Both panels in BED file format were downloaded from Oxford Nanopore Technologies Adaptive Sampling Catalogue [56] at the time the study was conducted. To our knowledge, the hereditary cancer panel is not available on the Adaptive Sampling Catalogue anymore, but details about the dataset can be found in the associated article [55]. In essence, the hereditary cancer panel contains the genomic coordinates of 147 cancer predisposition genes. This is the equivalent of approximately 16 Mb and covering 0.49% of the current reference genome (GRCh38/hg38), whereas the RRMS panel contains 311 Mb of regions that cover 7,186,000 CpG sites present in the DNA methylation landscape, including CpG islands, shores, shelves, promoters, enhancers, and genes. The merging of the two panels resulted in approximately 327 Mb, targeting 9.93% of the human genome (Supplementary Material S2).

### 4.4. DNA Library Preparation and Adaptive Sampling Sequencing

The DNA library was prepared with an optimized version of the Ligation sequencing gDNA V14-reduced representation methylation sequencing (RRMS) protocol (SQK-LSK114, ONT, Oxford, UK). Briefly, because the initial input of 2 µg of DNA was not enough to obtain relevant enrichment on the target regions, due to approximately 25% loss of DNA during the library preparation, we decided to start with an input of DNA ranging between 3 and 4 µg. By doing this we were able to achieve or be close to the 50–65 femtomoles of DNA library per load, as recommended in the adaptive sampling documentation [38]. Between each library step, DNA was quantified using NanoDrop ND-1000 and then loaded into a R10.4.1 flow cell (FLO-MIN114, ONT, Oxford, UK). The exact inputs and per load DNA amounts are provided in Supplementary Material S1, Table S5. The adaptive sampling sequencing was performed on a MinION Mk1B device connected via a Type -A USB to a computer with the Nvidia GeForce RTX 3080 TI card. The sequencing parameters were set in the MinKNOW software (v.6.0.14) as follows: adaptive sampling mode set to enrich the target regions from the combined RRMS and hereditary cancer panel, base calling off, and the run set to 96 h. After approximately 22 h and 45 h, flow cells were washed with the Flow Cell Wash Kit (EXP-WSH004, ONT, Oxford, UK) and reloaded for a total of 3 DNA library loads. Details regarding the sequencing runs can be found in Supplementary Material S1, Table S4.

### 4.5. Sequencing Data Processing and DNA Methylation Analysis

The POD5 files containing the raw data were base called and methylation called (5mC_5hmC) using Dorado’s (v.0.9.1) high accuracy mode (HAC) directly from the MinKNOW interface. The resulting modBAM files were merged and indexed using samtools (v.1.9). Subsequently, the BAM files were then aligned with minimap2 (version 2.28-r1221-dirty) to the same human reference genome (GRCh38/hg38) used in the construction of the adaptive sampling panel [57]. The processing step continued with calculating the DNA methylation frequencies, also known as β-values. For this modBAM files were converted to bedMethyl files using Modkit (v.0.4.4). The generated bedMethyl files contain the required data for the statistical analysis step in R, such as genomic coordinates (chr:n-n + 1), coverage, type of modification, and the β-value for each identified CpG. The bedMethyl files from all the samples were aggregated into one file from which a total of 64,390,996 CpG sites were identified. To increase the accuracy, a minimum of 5 reads per CpG site and no missing value (noNA) thresholds were applied, before dividing the aggregate file into three datasets based on modification: 5mC, 5hmC, and both modifications.

The ONT CpG sites datasets obtained earlier were compared with the CpG sites present in the Illumina Infinium Methylation Arrays, previously used for construction of Hannum’s [15], Horvath’s [16,49], and Weidner’s [17] clock. Because the two platforms use different CpG site annotations, we first converted the IlmnID to the genomic coordinates of the CpGs to make it the same format as the Nanopore datasets. Then we converted the genomic locations of the epigenetic clocks data from hg19 to hg38 for Horvath’s and Hannum’s clock, respectively, from hg18 to hg38 for Weidner’s clock using the LiftOver R function (Supplementary Material S1, Table S10). Genes overlapping with differentially methylated CpG sites were annotated based on genomic coordinates using the GenomicRanges package (v1.56.1). Genomic features were mapped to gene identifiers using the biomaRt package (v 2.60.1), which queries the Ensembl database to retrieve gene symbols, Entrez IDs, and corresponding transcript annotations. To investigate the functional implications of CpG sites, Gene Ontology (GO) enrichment analysis was performed using the clusterProfiler R package (v4.12.0). The GO enrichment analysis included terms from the biological process (BP), molecular function (MF), and cellular component (CC) categories. Enrichment was evaluated using enrichGO() from clusterProfiler. R functions and complete code including step by step instructions are available online in the GitHub repository: https://github.com/Independent-Research-Lab/nanopore-epigenetics (version 1, accessed on 11 May 2025).

### 4.6. Regression Models

Two regression methods were used in R to fit CpG beta values on known sample ageing points: A regularized regression model (Elastic Net) with cross-validation from the package glmnet (ver. 4.1-8) was used, with alpha = 0.5 (Lasso and Ridge regularization,), and respectively simple linear regression with function fit_row_lm. Statistical data were added as columns to the data frames and further used to analyze and graphical representation.

## Figures and Tables

**Table 1:** The ONT-CpG sites that overlapped with CpG sites from established epigenetic clocks.

ONT-CpG Site_Hg38	IllmnID	Overlapped with	Slope	R^2^	*p*-Value
chr1_1232655	cg19945840	Horvath353CpGs_hg18	0.0031	0.9303	0.0004
chr10_35605501	cg00168942	Horvath353CpGs_hg18	−0.0062	0.8008	0.0160
chr10_48465490	cg22796704	Horvath391CpGs_hg19	−0.0024	0.8664	0.0070
		Hannum71CpGs_hg18			
chr15_31483691	cg04875128	Horvath391CpGs_hg19	0.0019	0.8017	0.0158
		Hannum71CpGs_hg18			
chr15_51681722	cg16717122	Horvath391CpGs_hg19	0.0018	0.8589	0.0079
chr16_66697409	cg18693704	Horvath391CpGs_hg19	−0.0012	0.8488	0.0090
chr20_46029585	cg07547549	Hannum71CpGs_hg18	0.0046	0.8376	0.0105
		Horvath391CpGs_hg19			
chr6_30172367	cg03771840	Horvath391CpGs_hg19	0.0066	0.8947	0.0043
chr9_34662284	cg09722555	Horvath353CpGs_hg18	−0.0049	0.8165	0.0135

The table contains the 9 CpG sites identified with ONT that overlapped with Illumina CpG sites from 3 known epigenetic clocks. Their current genomic locations (hg38), matched Illumina IDs (hg18, hg19) and corresponding clocks are provided in the table. R^2^ = R-squared (correlation coefficient).

## Data Availability

The necessary instructions, R functions, and complete code to reproduce our analysis, including the download link for our modBAM input files for the seven blood samples, and our resulted data files, are available in our public GitHub repository: https://github.com/Independent-Research-Lab/nanopore-epigenetics.

## Supplementary Materials

The following supporting information can be downloaded at https://www.mdpi.com/article/10.3390/epigenomes9030025/s1. Supplementary Materials S1: Tables S1–S10. Supplementary Material S2: Bed file with adaptive sampling panel used in this study.
